# Boysenberry and apple juice concentrate reduced acute lung inflammation and increased M2 macrophage‐associated cytokines in an acute mouse model of allergic airways disease

**DOI:** 10.1002/fsn3.2119

**Published:** 2021-01-11

**Authors:** Odette M. Shaw, Roger D. Hurst, Janine Cooney, Gregory M. Sawyer, Hannah Dinnan, Sheridan Martell

**Affiliations:** ^1^ Nutrition & Health Group Food Innovation Portfolio The New Zealand Institute for Plant and Food Research Limited Palmerston North New Zealand; ^2^ Food Innovation Portfolio The New Zealand Institute for Plant and Food Research Limited Palmerston North New Zealand; ^3^ Biological Chemistry & Bioactives Group Food Innovation Portfolio The New Zealand Institute for Plant and Food Research Limited Hamilton New Zealand

**Keywords:** allergic airways inflammation, alternatively activated macrophages, anthocyanins, apple, Boysenberry

## Abstract

Bioactive compounds including anthocyanins and other polyphenols are associated with reduced lung inflammation and improved lung function in asthma and other lung diseases. This study investigated the effects of a Boysenberry and apple juice concentrate, high in cyanidin glycosides, ellagitannins, and chlorogenic acid, on a mouse model of allergic airways inflammation. Male C57BL/6J mice were orally gavaged with 2.5 mg/kg of total anthocyanins (TAC) from BerriQi® Boysenberry and apple juice concentrate (0.2 mg/kg human equivalent dose) or water control 1 hr before an acute intranasal ovalbumin (OVA) challenge and were gavaged again 2 days after the intranasal challenge. Consumption of BerriQi® Boysenberry and apple juice concentrate significantly decreased OVA‐induced infiltrating eosinophils, neutrophils, and T cells in the lung, and mucous production. Quantification of gene expression for arginase (Arg1), chitinase 3‐like 3 (Ym‐1), found in inflammatory zone (Fizz1), which have been associated with an anti‐inflammatory macrophage phenotype (M2), found significantly increased Arg1 expression in the lung in the Boysenberry and apple juice concentrate treatment group. There was also increased production of M2‐associated cytokines C‐X‐C motif chemokine ligand (CXCL) 10 and C‐C motif chemokine ligand (CCL) 4. These results suggest that consumption of BerriQi® Boysenberry and apple juice concentrate promoted a shift toward an anti‐inflammatory environment within the lung leading to reduced immune cell infiltration and tissue damage.

## Graphical abstract

We identified the polyphenols in a Boysenberry and apple juice concentrate and then investigated whether consumption inhibited immune cell infiltration into the lung in response to an allergen challenge in mice. We found Boysenberry and apple juice concentrate decreased immune cell infiltration and increased anti‐inflammatory genes and proteins suggesting that Boysenberry and apple juice concentrate mediated a switch to a more anti‐inflammatory lung environment leading to reduced tissue damage.

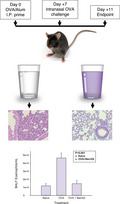

## 1 INTRODUCTION

Asthma is a heterogeneous, chronic, inflammatory lung disease characterized by reversible airways obstruction, bronchospasm, and infiltration of immune cells (Agache & Akdis, 2016; Agrawal & Bharadwaj, 2005; Barnes, 1996). It is estimated that 150 million people are affected by asthma worldwide, with a 5%–15% prevalence in children (WHO, 2003), and there is evidence that early life exposure to air pollution caused by vehicle exhaust, environmental dust, and industrial processes increases the severity of asthma in children (Jung et al., 2015; Hsu et al., 2015; Miller & Peden, 2014). The respiratory symptoms such as cough and wheeze are worsened by exposure to pollution (Hoek et al., 2012). Proinflammatory cytokine production in response to allergens by immune cells is further increased with concomitant pollution exposure (Acciani et al., 2013; Brandt et al., 2015; Carlsten et al., 2016; Kim et al., 2011; van Voorhis et al., 2013). Eosinophils, in particular, produce reactive oxygen species and cytokines, leading to epithelial damage and contribute to mucosal inflammation and the recruitment of other proinflammatory immune cells (Amin et al., 2016; Bossley et al., 2012; Brown et al., 1998; Trivedi & Lloyd, 2007). These repeated acute inflammatory responses lead to tissue damage and remodeling, contributing to airway hyperresponsiveness, mucus cell hyperplasia, fixed airway flow obstruction, and loss of lung function over time (Ahdieh et al., 2001; Al‐Muhsen et al., 2011; Bergeron et al., 2009; Brightling et al., 2012).

Large‐scale epidemiological studies have found that increased fruit and vegetable consumption correlates with reduced asthma symptoms (McKeever & Britton, 2004; Nurmatov et al., 2011; Okoko et al., 2007; Rosenlund et al., 2011). These dietary‐related improvements in lung function benefits are also seen in people living in polluted environments (Burbank et al., 2018; Pounis et al., 2018; Stevens et al., 2019). Fruits and vegetables contain numerous bioactive compounds, including anthocyanins and procyanidins, which have been shown to attenuate lung inflammation in cell and animal models of allergy and asthma (Coleman et al., 2016; Coleman & Shaw, 2017; Park et al., 2007; Sawyer et al., 2017; Shaw et al., 2016, 2017). Human population studies have identified that dietary intake of foods high in polyphenols and tetraterpenes (Pounis et al., 2018) such as apples, pears (Garcia‐Larsen et al., 2018), carrots, tomatoes (Rosenlund et al., 2011), and citrus is inversely correlated with the frequency and severity of reported asthma symptoms, especially wheezing and coughing (Garcia et al., 2005; Garcia‐Larsen et al., 2018; McKeever & Britton, 2004; Rosenlund et al., 2011). Previously, we have identified that Boysenberry consumption led to decreased chronic lung inflammation and improved lung tissue repair in an animal model of chronic allergic lung inflammation (Shaw et al., 2016). Boysenberries contain high concentrations of anthocyanins (261 mg/g), ellagitannins, and other polyphenols (241 mg/g) (Cooney et al., 2004; Ghosh et al., 2006; McGhie et al., 2012). Apple contains approximately 120–200 mg/g total polyphenols (Paturi et al., 2014), and we have found that procyanidin‐enriched apple extracts suppressed IL‐4‐mediated cytokine production in cell culture models of lung epithelial allergic inflammation (Coleman et al., 2016; Sawyer et al., 2017).

There is increasing interest in understanding the mechanisms of action that specific plant bioactives have in the human body. This is partially to better understand the benefits of consuming specific fruits and vegetables and partially to add value to specific foods through validated health claims. There is also interest in determining if combining specific plants containing different polyphenols can augment the health benefits above those seen with the individual plant. Use of animal models, where dietary intake can be tightly controlled, is useful for both demonstrating/revealing the efficacy for identified compounds and determining the biological mechanisms of action. The aim of this study was to determine whether the combination of Boysenberries and apple, as found in BerriQi® Boysenberry and apple juice concentrate at a dose of 2.5 mg/kg total anthocyanins (TAC), could reduce allergic airways inflammation in response to acute ovalbumin (OVA) exposure in a mouse model system. We also sought to determine the mechanisms involved in any ameliorating effect.

## 2 MATERIALS AND METHODS

### 2.1 Mice and materials

C57BL/6J male mice were group housed on 12‐hr light/dark cycle in a conventional animal facility at The New Zealand Institute for Plant and Food Research Limited (Palmerston North, New Zealand). Mice were fed Prodiet RMH1800 standard chow for rodents (Lab Diet) and filtered water ad libitum throughout the study, and all attempts to minimize suffering were made. All experimental procedures were approved by the AgResearch Grasslands Animal Ethics Committee (AE approvals #14839, #14731 and #14016) and carried out in accordance with the Animal Welfare Act (1999). A commercially available Boysenberry and apple juice concentrate ingredient (BerriQi®) was supplied by and is available from Anagenix Ltd (Auckland, New Zealand). Legendplex™ 13‐plex Th cytokine, proinflammatory cytokine, and proinflammatory chemokine panels, Zombie NIR™ fixable viability dye, and anti‐mouse CD3 (clone 17A2), CD4 (clone GK1.5), CD8a (clone 53‐6.7), CD80 (clone 16‐10A1), CD86 (clone GL‐1), CD11c (clone N418), CD45 (clone 30‐F11), CD206 (clone C068C2), CD14 (clone Sa14‐2), Ly6C (clone HK1.4), Gr‐1 (clone RB6‐8C5), I‐A/I‐E (MHC class II; clone M5/114.15.2), and F4/80 (clone BM8) were purchased from BioLegend (San Diego, CA, USA). Anti‐mouse SiglecF (clone E50‐2440) and CD11b (clone M1/70) were from BD Biosciences. Ovalbumin (OVA), and Alum were purchased from Sigma (Auckland, New Zealand). iScript Advanced cDNA kit was from Bio‐Rad Laboratories (Hercules, CA, USA) and 2X LightCycler® 480 SYBR Green I Master mix was from Roche Diagnostics NZ. Taqman® Gene Expression Master Mix probe sets for murine inducible nitric oxide synthase (Nos2) (Mm00440502_m1), chitinase 3‐like 3 (Ym‐1/Chil3) (Mm00657889_mH), found in inflammatory zone (Fizz1/Retnla) (Mm00445109_m1) and arginase (Arg1) (Mm00475988_m1) genes were purchased from Thermo Fisher Scientific NZ (Auckland, NZ), and oligonucleotide primers for murine β‐actin and GAPDH housekeeping genes were synthesized by Integrated DNA Technologies. Unless otherwise stated, all cell culture media, supplements, and buffers were purchased from Life Technologies NZ.

### 2.2 Boysenberry and apple juice concentrate chemical composition analysis

BerriQi®, a mixture of Boysenberry juice concentrate sourced from Boysenberries New Zealand and apple juice concentrate sourced from Profruit Limited, was blended in a proprietary ratio by Anagenix Ltd. The polyphenol content of the BerriQi® Boysenberry and apple juice concentrate was determined by liquid chromatography‐mass spectrometry (LC‐MS) using an LTQ linear ion trap mass spectrometer fitted with an ESI interface (Thermo Fisher Scientific) coupled to an Ultimate 3000 UHPLC and PDA detector (Dionex). A weighed quantity of the concentrate was dissolved in 0.1% formic acid(aq) to give an aqueous solution of concentration of 20 mg/ml. For quantification of anthocyanins, the sample was diluted 5 times further to ensure analyte concentrations were within the linear calibration range of the PDA and MS detectors. For quantification of nonanthocyanin phenolics, both diluted and undiluted samples were analyzed. Anthocyanins were separated on a Poroshell 120 SB‐C18, 2.1 × 150 mm, 2.7 µm, analytical LC column (Agilent), maintained at 70°C. The solvents were (A) 5:3:92 acetonitrile:formic acid:water v/v/v and (B) acetonitrile + 0.1% formic acid (flow rate, 200 µl/min). The initial mobile phase, 100% A, was held for 2 min before being ramped linearly to 88% A at 14 min, returning to 5% A at 15 min, and held for 4 min before resetting to the original conditions. The sample injection volume was 10 µl. The MS data were acquired in the positive mode. Standards of cyanidin‐3‐O‐glucoside were used to quantitate anthocyanin concentrations with PDA detection at 520 nm, and the results for individual and total anthocyanin concentrations are reported as cyanidin‐3‐O‐glucoside equivalents.

Other phenolic compound separation was achieved using a Hypersil GOLD aQ 1.9 µ C18 175 Å (Thermo Scientific), 150 × 2.1 mm column maintained at 45°C. The solvents were (A) water + 0.1% formic acid and (B) acetonitrile + 0.1% formic acid (flow rate, 200 µl/min). The initial mobile phase, 95% A/5% B, was ramped linearly to 85% A at 10 min, held for 3.75 min, then ramped linearly to 75% A at 18 min, 67.2% A at 25 min, 50% A at 28 min, and 3% A at 29 min, and held for 4 min before resetting to the original conditions. The sample injection volume was 4 µl. The MS data were acquired in the negative mode. The phenolic acids, gallic acid, ellagic acid, protocatechuic acid, chlorogenic acid (3‐p‐caffeoylquinic acid) and caffeic acid, the flavan‐3‐ols, catechin and epicatechin, the procyanidin B2, the nonglycosylated flavonols, quercetin and myricetin, and the chalcone, phloretin‐2‐O‐glucoside were quantified by LC‐MS using pure standards of these compounds. Detected derivatives of coumaric acid are expressed as coumaric acid equivalents. Detected flavonol glycosides were quantified by LC‐MS using a pure standard of quercetin‐3‐O‐glucoside and are expressed as quercetin‐3‐O‐glucoside equivalents. Other detected chalcones were quantified as phloretin‐2‐O‐glucoside equivalents. Hydrolyzable tannins were quantified by LC‐MS using a standard of sanguiin H6 that had been isolated previously (>98% purity by LC‐MS). Other detected tannins were quantified as sanguiin H‐6 equivalents.

### 2.3 Ovalbumin‐induced airway inflammation model

Allergic airway disease was induced as previously described (Shaw & Harper, 2013; Shaw et al., 2017). For the Boysenberry and apple interventions, mice were randomized into receiving either water (vehicle control) or 2.5 mg/kg TAC in the BerriQi® Boysenberry and apple juice concentrate as previously described (Shaw et al., 2017). Briefly, mice were fasted for 4 hr before being orally gavaged with water (control) or at a dose of 2.5 mg/kg body weight TAC in the BerriQi® Boysenberry and apple juice concentrate made up to a total volume of 200 µl in water 1 h before OVA challenge and again 2 days postchallenge. Mice were sacrificed by anesthetic overdose 4 days following intranasal ovalbumin challenge and immune parameters, and gene expression was analyzed.

### 2.4 Immune parameter analysis

Bronchoalveolar lavage fluid (BALF) and lung tissues were collected as previously described, and immune cells were phenotyped by flow cytometry (Shaw & Harper, 2013). Lung tissue supernatant for cytokine analysis was prepared as previously described (Shaw et al., 2017). Cytokine production in lung tissue supernatant was measured by Legendplex bead‐based multiplex immunoassays as per the manufacturer's instruction. Both cell phenotyping and the cytokine multiplex assays were analyzed using a BD FACSverse (BD Biosciences). H&E and AB‐PAS histological staining were performed by Massey IVABS histology unit.

### 2.5 Real‐time qPCR analysis

Mouse lung tissue was snap‐frozen in liquid nitrogen and crushed into powder using a mortar and pestle with liquid nitrogen to preserve RNA integrity. The RNA was extracted from the powder using a TRIzol total RNA extraction protocol. RNA was quantified using an LVis plate in a POLARstar Omega plate reader (BMG Labtech), and the quality of the ribosomal RNA bands confirmed by agarose gel electrophoresis (data not shown). cDNA was synthesized from the lung sample RNA templates using the iScript™ cDNA Synthesis kit as per the manufacturer's instructions. Taqman® Gene Expression Assays for each gene of interest (Arg1, Nos2, Ym‐1, and Fizz1) were performed as per the manufacturer's protocols. Two housekeeping genes, GAPDH (forward primer sequence: GTTGTCTCCTGCGACTTCA; reverse primer sequence: GGTGGTCCAGGGTTTCTTA) and β‐actin (forward primer sequence: CTGTCCCTGTATGCCTCTG; reverse primer sequence: ATGTCACGCACGATTTCC) (Xiang et al., 2012), were used as controls to determine the differential gene expression and were amplified using the LightCycler® 480 SYBR Green master mix as per the manufacturer's instructions. All genes of interest and both housekeeping genes were amplified in quadruplicate for each lung sample using a Bio‐Rad™ CFX384™ Real‐Time PCR Detection System. Normalized (ΔΔCq) gene expression was performed using the Bio‐Rad CFX Manual 3.1 software. The two housekeeping genes were identified as reference genes within the software which allowed the gene expression data from each group to be expressed as a fold change relative expression to the naïve group.

### 2.6 Statistical analysis

All data were analyzed using one‐way analysis of variance (ANOVA) with a Tukey's post hoc test and graphed in SigmaPlot 12.5 (Systat Software Inc.).

## 3 RESULTS

### 3.1 Chemical composition of the Boysenberry and apple juice concentrate

The results of the LC‐MS analysis showed that cyanidin glycosides, ellagitannins, and chlorogenic acid were the major components in BerriQi® Boysenberry and apple juice concentrate (Table 1, Figures S1, S2). Minor components included phloretin 2‐*O*‐glucoside and a mix of phenolic acids, flavonol glycosides, flavanol monomers, and procyanidins. The major classes of phenolic compounds were anthocyanins (1969 μg/mL) and hydrolyzable tannins (946 μg/mL), accounting for 56% and 27%, respectively, of the total phenolics quantified. The most abundant tannins were ellagic acid (449 μg/mL) and sanguiin H6 (213 μg/mL).

**TABLE 1:** Phenolic compounds detected in BerriQi® (µg/mL)

Peak	M^+^	(M‐H)^‐^	Compound	µg/mL	µg/g DW
			Anthocyanins		
1	611		Cyanidin 3‐*O*‐sophoroside	883	937
2	449		Cyanidin 3‐*O*‐glucoside	571	606
3	481		Cyanidin 3‐*O*‐sambubioside	24	25
4	757		Cyanidin 3‐*O*‐(2‐glucosylrutinoside)	411	436
5	595		Cyanidin 3‐*O*‐rutinoside	62	66
6	727		Cyanidin 3‐*O*‐xylosylrutinoside	18	20
			Phenolic acids		
7		169	Gallic acid	140	149
8		153	Protocatechuic acid	35	37
10		353	Chlorogenic acid	69	73
11		179	Caffeic acid	7	7
15		337	4‐*p*‐Coumaroylquinic acid	26	28
17		337	5‐*p*‐Coumaroylquinic acid	3	3
			Flavan−3‐ols and procyanidins		
9		335^a^	Catechin	3	3
12		577	Procyanidin B2	6	6
13		335^a^	*Epi*catechin	21	22
25		579	Unknown procyanidin isomer	3	3
			Hydrolyzable tannins		
14		1567	Sanguiin H10 isomer 1	9	10
16		469	Sanguisorbic acid dilactone	120	127
19		2036.5	Galloyl‐SH6	66	70
20		1567	Sanguiin H10 isomer 2	61	65
21		2501	Lambertian C (minus ellagic acid)	11	12
22		2803	Lambertian C	17	18
24		1869	Sanguiin H6	213	226
26		301	Ellagic acid	449	477
			Flavonols		
27		609	Quercetin 3‐*O‐*rutinoside	6	6
28		463	Quercetin 3‐*O‐*galactoside	17	18
29		477	Quercetin 3‐*O‐*glucuronide	36	38
30		463	Quercetin 3‐*O‐*glucoside	13	14
31		433	Quercetin 3‐*O‐*pentoside 1	9	10
32		433	Quercetin 3‐*O‐*pentoside 3	6	6
33		433	Quercetin 3‐*O‐*pentoside 2	13	14
35		447	Quercetin 3‐*O‐*rhamnoside	15	16
37		301	Quercetin	19	20
			Chalcones		
34		567	Phloretin 2‐*O‐*xylo‐glucoside	9	10
36		481^a^	Phloretin 2‐*O‐*glucoside	62	66
			Unknowns		
18		563^a^	unknown	71	75
23		639	unknown	22	23
			Total	1557	1653

Note

M^+^ and (M‐H)^‐^ ions are the pseudomolecular ions used for identification of compounds by liquid chromatography‐mass spectrometry (LC‐MS). All identifications confirmed by MS/MS^n^ experiments. Peak numbers refer to chromatograms shown in supplementary data.

^a^ Detected as [M + formate]^‐^ adduct.

### 3.2 Effect of Boysenberry and apple juice concentrate intervention on ovalbumin‐induced allergic airways inflammation

**FIGURE 1:**
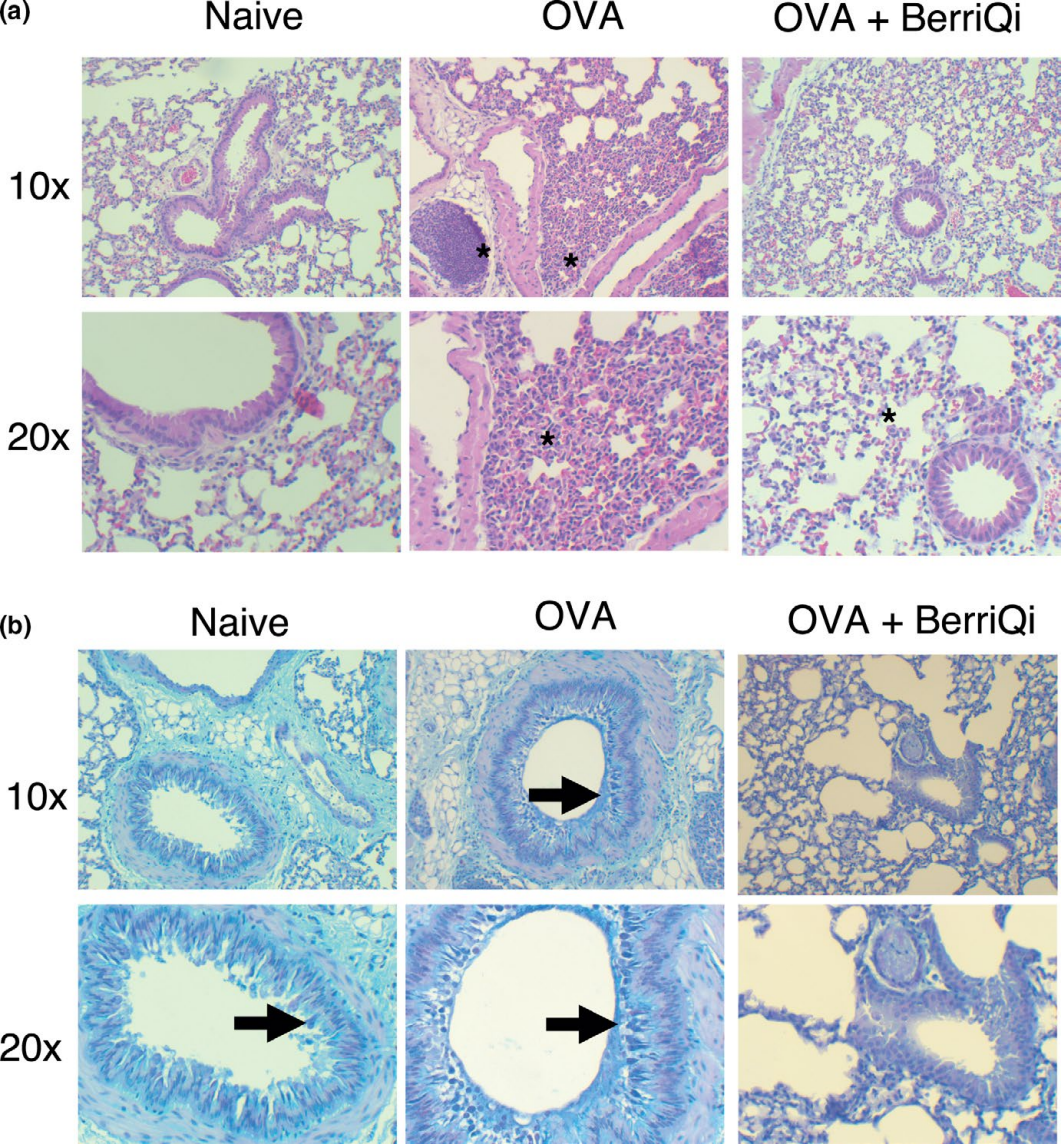
BerriQi® Boysenberry and apple juice concentrate suppresses ovalbumin‐induced airway inflammation. (a) Representative hematoxylin and eosin stained lung tissue from naïve, OVA‐challenged and OVA‐challenged mice treated with BerriQi® Boysenberry and apple juice concentrate. Magnification 10x (top) and 20x (bottom) Asterisk = cell infiltration. (b) Representative Alcian‐blue Periodic acid‐Schiff stained lung tissue from naïve, OVA‐challenged and OVA‐challenged mice treated with BerriQi® Boysenberry and apple juice concentrate. Magnification 10x (top) and 20x (bottom). Arrow = mucous producing goblet cells

**FIGURE 2:**
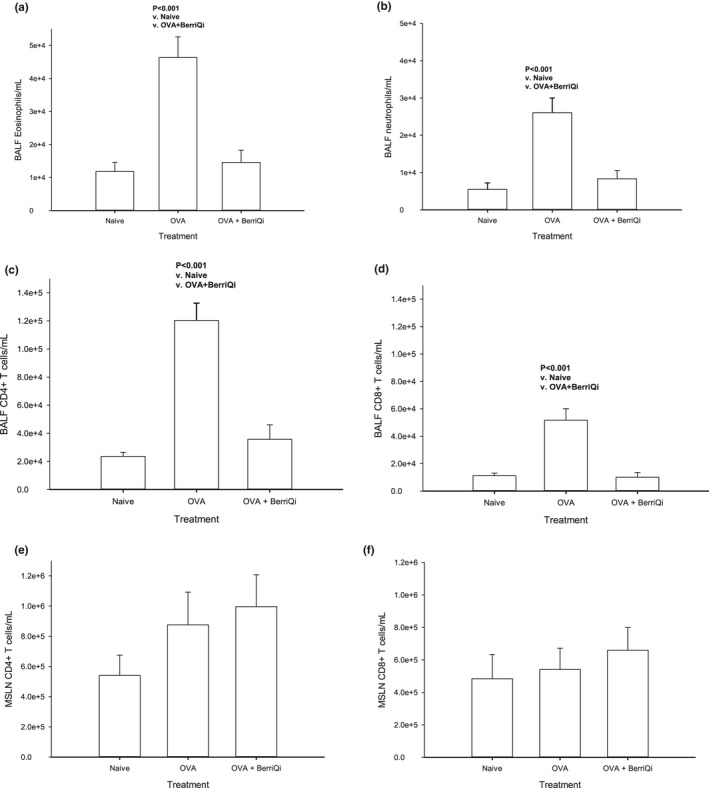
BerriQi® Boysenberry and apple juice concentrate suppresses ovalbumin‐induced immune cell infiltration into the lung. (a) Total eosinophil, (b) total neutrophil, (c) CD4 + T cells, and (d) CD8 + T cells in bronchoalveolar lavage fluid (BALF), and (e) CD4 + and (f) CD8 + T cells number in the mediastinal lymph node (MSLN), were determined 4 days post‐OVA challenge. Data presented as mean ± *SEM* *p* < .001 compared with naïve and OVA challenge + BerriQi® Boysenberry and apple juice concentrate (one‐way ANOVA with Tukey's post hoc test) for two experimental replicates with *n* = 9–10 per treatment groups

Acute intranasal OVA exposure resulted in an infiltration of immune cells into the lung (Figure 1a) and increased mucous production (Figure 1b). Consumption of 2.5 mg/kg TAC BerriQi® Boysenberry and apple juice concentrate reduced the infiltration of immune cells and decreased OVA‐induced mucous production (Figure 1a,b). We quantified the type and number of immune cells infiltrating into the lung and found that acute OVA exposure significantly increased (*p* < .001) infiltrating eosinophils (CD45+/CD11b+/SiglecF+), neutrophils (CD45 + Ly6C+Gr‐1+), and T cells (CD45+/CD3+/CD4 + or CD45+/CD3+/CD8a+), compared with the lung of naïve animals (Figure 2a‐d). Compared with animals only exposed to OVA, those that also consumed 2.5 mg/kg TAC BerriQi® Boysenberry and apple juice concentrate showed a significant decrease (*p* < .001) in the number of infiltrating eosinophils, neutrophils, and T cells in the lung (Figure 2a‐d). We saw no change in the number of CD4 + or CD8 + T cells in the mediastinal (lung draining) lymph node for any of the treatment groups (Figure 2e‐f).

**FIGURE 3:**
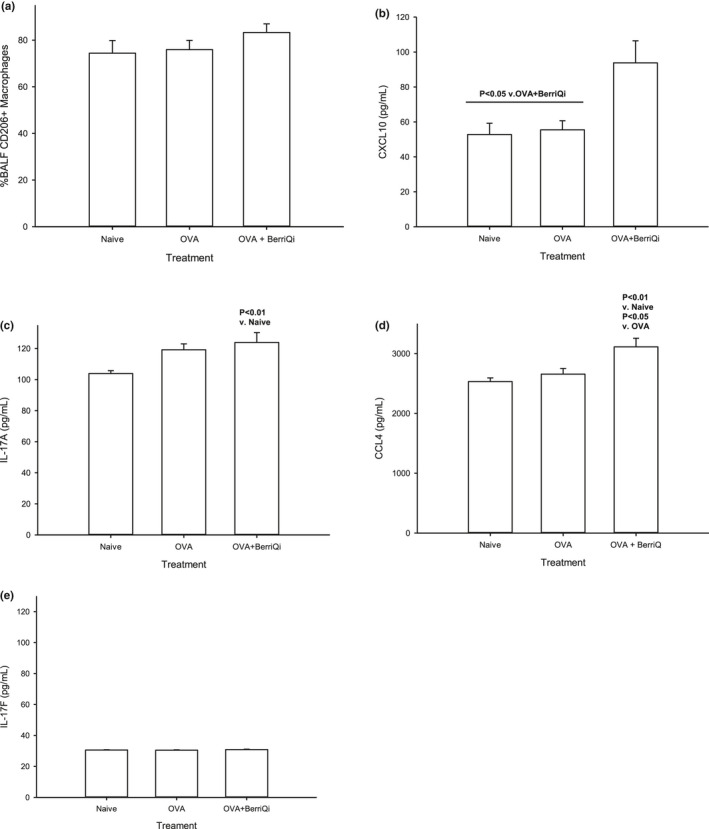
BerriQi® Boysenberry and apple juice concentrate increased CD206 + macrophage and IL‐17A, CXCL10, and CCL4 cytokine concentrations. (a) Percentage of CD206 + macrophages in BALF, and lung tissue production of (b) CXCL10, (c) IL‐17A, (d) CCL4, and (e) IL‐17F was determined 4 days post‐OVA challenge by Legendplex. Data presented as mean ± *SEM*, *p* < .05 compared with OVA challenge + BerriQi® Boysenberry and apple juice concentrate, *p* < .01 compared with naïve and OVA challenge (one‐way ANOVA with Tukey's post hoc test) for two experimental replicates with *n* = 10 per treatment groups

There was a trend toward an increased percentage of CD206+/CD14‐ macrophages in the lungs of mice that consumed 2.5 mg/kg TAC BerriQi® Boysenberry and apple juice concentrate (Figure 3a). To determine whether it was possible that there was increased alternatively activated macrophages, we measured the gene expression of Arg1, Ym‐1, Fizz1, and Nos2 in lung tissue. We found that both OVA alone and 2.5 mg/kg TAC BerriQi® Boysenberry and apple juice concentrate consumption led to a significant (*p* < .01) fold increase in Ym‐1 (4.0 ± 2.4 and 4.7 ± 1.8, respectively) and Fizz1 (15.4 ± 11.6 and 23.0 ± 13.6, respectively) gene expression compared to naïve mice (Table 2). 2.5 mg/kg TAC BerriQi® Boysenberry and apple juice concentrate also led to a significant increase in Arg1 compared to OVA alone (*p* < .05) and naïve (*p* < .001), whereas OVA alone did not significantly increase Arg1 compared to naïve mice (Table 2). We found no significant fold change in Nos2 gene expression between any of the treatment.

**TABLE 2:** BerriQi® Boysenberry and apple juice concentrate increases alternatively activated macrophage gene expression in the lung

Target Gene	Naïve (*n* = 9)	OVA (*n* = 9)	OVA + BerriQi (*n* = 10)
Arg1	1 (0.66)	9.0 (7.3)	18.9 (12.4)**^,†^
Ym−1	1 (0.7)	4.0 (2.4)**	4.7 (1.8)**
Fizz1	1 (0.8)	15.4 (11.6)**	23.0 (13.6)**
Nos2	1 (0.6)	1.0 (0.5)	1.2 (0.5)

Note

Mean fold change (*SEM*) in gene expression was measured by real‐time qPCR in lung tissue 4 days post‐OVA challenge.

**p < .01 compared with naïve, ^†^ *p* < .05 compared with OVA alone (one‐way ANOVA with Tukey's post hoc test) for 4 experimental replicates with *n* = 9–10 per treatment groups.

**FIGURE 4:**
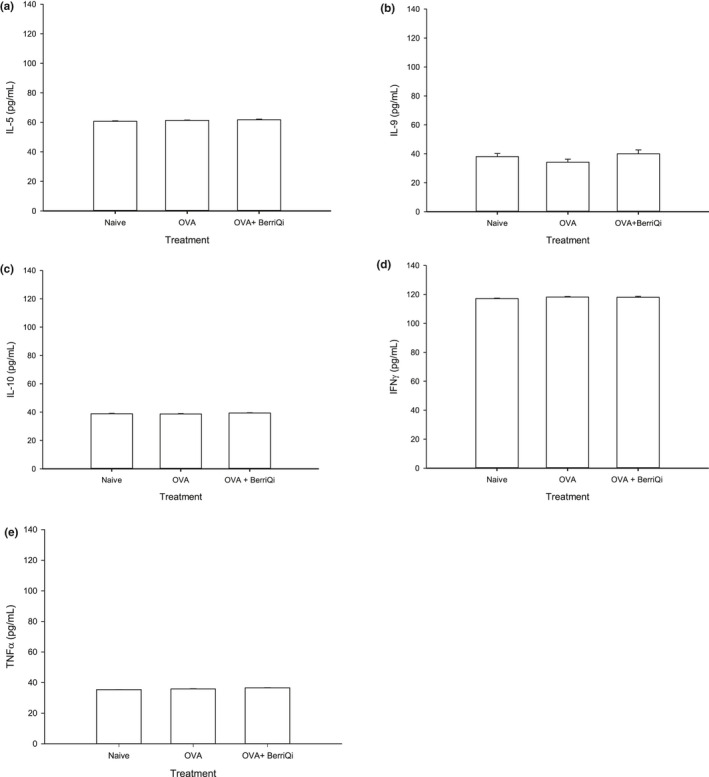
BerriQi® Boysenberry and apple juice concentrate does not alter classical Th‐1/Th‐2 cytokines. Lung tissue production of (a) IL‐5, (b) IL‐9, (c) IL‐10 (d) IFNγ, and (e) TNFα were determined 4 days post‐OVA challenge by Legendplex. Data presented as mean ± *SEM* for two experimental replicates with *n* = 9–10 per treatment groups

Consumption of 2.5 mg/kg TAC BerriQi® Boysenberry and apple juice concentrate led to increased levels of the cytokines interleukin (IL)‐17A, C‐X‐C motif chemokine ligand (CXCL)10, and C‐C motif chemokine ligand (CCL)4 (Figure 3b‐d) 4 days following OVA challenge, but did not affect the IL‐17F concentration (Figure 3e). We saw no effect on the concentrations of the classical Th‐1/Th‐2 cytokines interferon gamma (IFNγ), tumor necrosis factor alpha (TNFα), IL‐5, IL‐9, or IL‐10 in either the BerriQi® Boysenberry and apple juice concentrate treated or the OVA alone mice compared to naïve controls (Figure 4a‐e), and the concentration of IL‐4 and IL‐13 was below the detection limits of the assay for all treatment groups.

## 4 DISCUSSION

We evaluated the effects of dietary supplementation with 2.5 mg/kg TAC BerriQi® Boysenberry and apple juice concentrate, on the immune responses in a mouse model of acute allergic airways inflammation. Our results show that consumption of 2.5 mg/kg TAC BerriQi® Boysenberry and apple juice concentrate reduced granulocyte and local T‐cell infiltration into the lung after OVA challenge, but did not alter T‐cell activation within the lung draining lymph node or the levels of classical Th‐2 and Th‐1 cytokines in the lung at four days following OVA challenge. Our current results indicated that BerriQi® Boysenberry and apple juice concentrate had little impact on the Th‐2/Th‐1 mediated allergic response of mice, but rather targeted innate proinflammatory immune pathways. This is consistent with our previously reported finding in a mouse model of chronic allergic airways inflammation using 10 mg/kg TAC Boysenberry juice concentrate (Shaw et al., 2016). Chemical composition analysis showed that the BerriQi® Boysenberry and apple juice concentrate formulation contained high concentrations of cyanidin glycosides, ellagitannins, and chlorogenic acid. These compounds have been previously shown to reduce inflammatory signaling in vitro (Cassidy et al., 2015; El‐Shitany et al., 2014; McGhie et al., 2012) and in vivo animal models of inflammation (Auclair et al., 2008; Denis et al., 2016; Guan et al., 2017; Impellizzeri et al., 2015; Shaw et al., 2017). Our current results suggest that consumption of 2.5 mg/kg TAC BerriQi® Boysenberry and apple juice concentrate, which also contains high levels of ellagitannins and chlorogenic acid, could have broader lung health benefits beyond allergic asthma disease by promoting the resolution of inflammation caused by innate immune cell overactivation.

Consumption of BerriQi® Boysenberry and apple juice concentrate had less of an effect on monocyte/macrophage infiltration into the lung than on granulocyte infiltration, and there was increased percentage of CD206 + monocytes. This suggested that there could be a shift to an alternatively activated macrophage (M2‐like) phenotype. To confirm this shift, we measured the changes in gene expression for Arg1, Ym‐1, and Fizz1, classic genes for identifying alternatively activated macrophages (Chung et al., 2016; Kurowska‐Stolarska et al., 2009) as well as Nos2, a proinflammatory gene closely associated with classically activated macrophages (M1‐like). We found that both OVA alone and with BerriQi® Boysenberry and apple juice concentrate consumption resulted in a significant fold increase in Ym‐1 and Fizz1 gene expression compared to naïve mice at 4 days following challenge, and we did not see increased Nos2 gene expression in any of the groups. These results suggested that 4 days post‐OVA challenge the infiltrating monocyte/macrophages were more M2‐like rather than M1‐like. This is consistent with other studies that have shown that lung macrophages express increased M2‐associated genes following OVA challenge (Siddiqui et al., 2013). Alternatively activated macrophage Fizz1 expression, in particular, has been associated with regulating Th‐2‐mediated lung inflammation by modulating IL‐4 and IL‐5 (Nair et al., 2009). It has also been shown that depletion of alternatively activated macrophages does not ameliorate allergic airways inflammation (Nieuwenhuizen et al., 2012).

However, BerriQi® Boysenberry and apple juice concentrate led to a significant increase in Arg1 compared to both OVA alone and naïve and OVA alone did not significantly increase Arg1 compared to naïve mice. Previous studies have shown that changes in Fizz1 and Ym‐1 gene expression can be constitutive in M2‐like macrophages (Wong et al., 2010), and arginase activity regulation has been shown to be regulated independently of Fizz1 and Ym‐1 gene expression in lung macrophages (Raes et al., 2002). Arginase expression, particularly by M2‐like macrophages, has been associated with lung remodeling (Martinez et al., 2009), and increased arginase activity is associated with lower iNOS activity through substrate competition, leading to reduced inflammation (Hey et al., 1997; Johann et al., 2007; Mori & Gotoh, 2000). The increase in Arg1 gene expression is similar to our previously reported study showing increased arginase protein expression by alternatively activated macrophages as a result of chronic Boysenberry consumption (Shaw et al., 2016). Further, research looking at an animal model Th‐2‐mediated inflammation has identified M2 macrophage‐derived Fizz1 as a key limiting factor for Th2‐mediated pulmonary inflammation (Nair et al., 2009).

Consistent with Boysenberry and apple juice concentrate polyphenols resulting in a greater shift to an anti‐inflammatory environment, mice that consumed 2.5 mg/kg TAC BerriQi® Boysenberry and apple juice concentrate showed increased levels of the cytokines CXCL10 and CCL4, which are produced by M2 macrophages, compared to OVA alone and naïve mice. CXCL10 and CCL4 are chemokines that attract monocytes/macrophages, and CXCL10 has been shown to also inhibit the infiltration of eosinophils in response to allergic airways inflammation (Su et al., 2008). IL‐17A was also increased in the BerriQi® Boysenberry and apple juice concentrate group compared to naïve mice only, and the levels of IL‐17F were not affected by any of the treatments at the time point measured. High IL‐17 expression, including IL‐17A and IL‐17F, has been implicated in asthma pathogenesis (Gurczynski & Moore, 2018; Wang & Liu, 2008). However, there is also some evidence that elevated IL‐17A (Linden & Dahlen, 2014) increases the abundance of MMP‐9, an important tissue remodeling protein in asthma (Shaw et al., 2016) and late‐stage increases in IL‐17A concentration can induce apoptosis of neutrophils and eosinophils (Linden & Dahlen, 2014; Wang & Liu, 2008). However, it not clear whether the increased IL‐17A seen in the BerriQi® Boysenberry and apple juice concentrate treatment is proinflammatory or anti‐inflammatory as there is no statistical difference between the OVA alone group, and further studies to determine the role of IL‐17A are needed. The reduced number of eosinophils and neutrophils seen with consumption of the BerriQi® Boysenberry and apple juice concentrate could be as a result of either a late‐stage IL‐17A increase causing granulocyte apoptosis or CXCL10‐mediated inhibition of granulocyte infiltration or a combination of the two factors. It is not yet clear how important these cytokines are for mediating the effects of BerriQi® Boysenberry and apple juice concentrate, and more work is needed to fully determine whether these cytokines are responsible for the decreased inflammatory response to OVA seen in this model and if alternatively activated (M2) macrophage are the cytokine source.

A switch to a more anti‐inflammatory M2 macrophage phenotype may be through the Boysenberry and apple polyphenols identified in the BerriQi® Boysenberry and apple juice concentrate directly inhibiting proinflammatory pathways. Ellagitannins have been shown in cell culture and animal models of chronic inflammatory diseases to reduce proinflammatory prostaglandins (Karlsson et al., 2010) cytokines, (Cornelio Favarin et al., 2013; Guan et al., 2017) and other proteins (El‐Shitany et al., 2014; Marin et al., 2013; Saba et al., 2013). Anthocyanins have also been shown to inhibit proinflammatory proteins (Esposito et al., 2014; Fu et al., 2014) and activate anti‐inflammatory pathways in models of inflammation (Chen et al., 2016; Edirisinghe et al., 2011; Khanna et al., 2001; Koh et al., 2015; Liu et al., 2015). Previously, we reported that 10 mg/kg TAC Boysenberry juice concentrate can increase the abundance of alternatively activated (M2) macrophages, which promote tissue repair in a chronic model of airways inflammation (Shaw et al., 2016). It is possible that the combination of the different polyphenols in the BerriQi® Boysenberry and apple juice concentrate acts on a number of different immune pathways to regulate the immune responses to OVA.

We found that mice that consumed BerriQi® Boysenberry and apple juice concentrate had reduced immune cell infiltration in response to acute OVA challenge, and this could be as a result of a shift toward an anti‐inflammatory environment within the lung. These results highlight the potential of anthocyanin‐rich Boysenberry and apple dietary supplementation to modulate innate immune pathways during acute allergic lung inflammation. Further work is needed to determine whether these pathways are also altered in other lung inflammatory conditions, such as air pollution exposure, and to determine the underlying molecular mechanisms the mediate the reported effects as well as clinical studies to show if these findings are translatable to human health.

## CONFLICT OF INTEREST

None of the other authors declare any other Competing Interest.

## AUTHOR CONTRIBUTIONS

O.M.S. designed, performed, analyzed, and interpreted the in vivo studies and wrote and edited the manuscript. J.C. performed, analyzed, and interpreted the chemical composition experiments. G.M.S. performed, analyzed, and interpreted in vivo studies, and both contributed to the writing and editing of the manuscript. H.D. and S.M. performed the in vivo studies and helped edit the manuscript. R.D.H. designed and directed the overall research program and helped edit the manuscript.

## ETHICAL REVIEW

All animal experimental procedures were approved by the AgResearch Grasslands Animal Ethics Committee (AE approvals #14839, #14731 and #14016) and carried out in accordance with the New Zealand Animal Welfare Act (1999).

## ETHICAL STATEMENTS

O.M.S. and R.D.H. report that they are named on a patent application related to the formulation of BerriQi® Boysenberry and apple juice concentrate. The title is “BOYSENBERRY, APPLE, AND BLACKCURRANT COMPOSITIONS AND METHODS OF PREPARATION AND USE THEREFOR,” PCT Application No. WO2019031972, dated 08 August 2018. The authors have not received any financial compensation nor will receive any personal royalty payments as a result of this.

## Supporting information

Fig S1‐S2
