# Supplementary material for: Boysenberry and apple juice concentrate reduced acute lung inflammation and increased M2 macrophage‐associated cytokines in an acute mouse model of allergic airways disease
Source: Food Sci Nutr. 2021 Jan 11;9(3):1491–503. doi: 10.1002/fsn3.2119 (PMC7958577; doi:10.1002/fsn3.2119)
Supplement: Supplementary file 1 — Fig S1‐S2 [file FSN3-9-1491-s001.docx]

**Supporting Information –Chemistry**

**S1 fig: Liquid Chromatography-Mass Spectrometry (LC-MS) anthocyanin chromatogram**
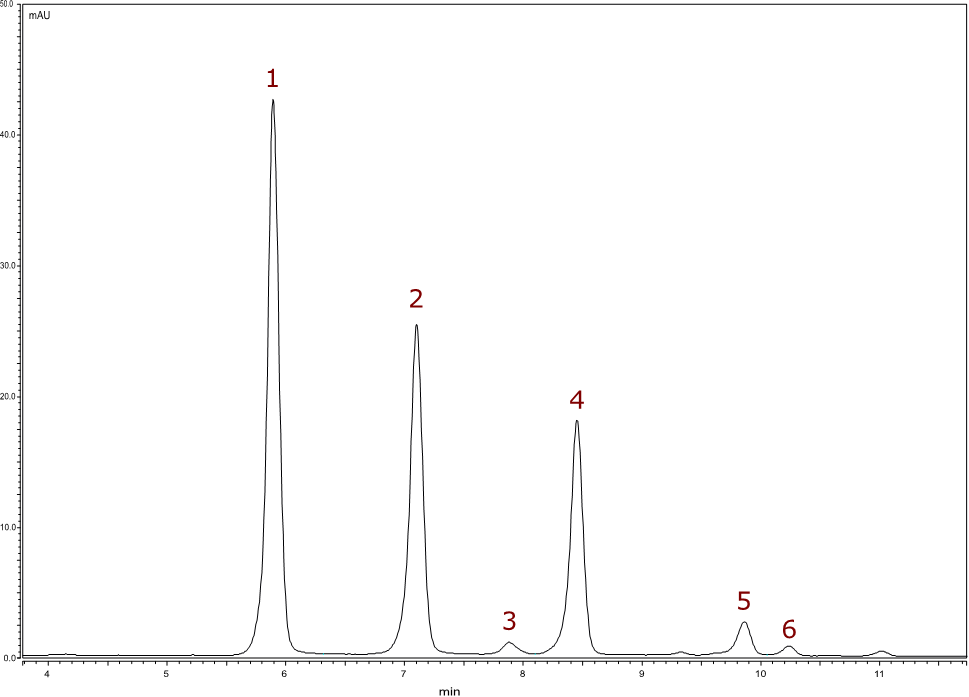


LC-MS chromatogram for BerriQi® concentrate, showing the anthocyanin UV/VIS profile measured at 520 nm. Peak numbers refer to compounds listed in Table 1.

**S2 fig: Liquid Chromatography-Mass Spectrometry (LC-MS) phenolic detection chromatograms**

LC-MS chromatograms for BerriQi® concentrate, showing PDA (photodiode array) phenolic profiles measured at 200–600 nm, 280 nm and 360 nm. Peak numbers refer to compounds listed in Table 1. X, denotes sorbic acid, a preservative added to the BerriQi concentrate during formulation. This was present at a concentration of ~2000 µg/mL.
